# Hansel, Gretel, and the Consequences of Failing to Remove Histone Methylation Breadcrumbs

**DOI:** 10.1016/j.tig.2019.12.004

**Published:** 2020-01-29

**Authors:** Teresa W. Lee, David J. Katz

**Affiliations:** 1 Department of Cell Biology, Emory University, Atlanta, GA 30322, USA

## Abstract

Like breadcrumbs in the forest, cotranscriptionally acquired histone methylation acts as a memory of prior transcription. Because it can be retained through cell divisions, transcriptional memory allows cells to coordinate complex transcriptional programs during development. However, if not reprogrammed properly during cell fate transitions, it can also disrupt cellular identity. In this review, we discuss the consequences of failure to reprogram histone methylation during three crucial epigenetic reprogramming windows: maternal reprogramming at fertilization, embryonic stem cell (ESC) differentiation, and the continuous maintenance of cell identity in differentiated cells. In addition, we discuss how following the wrong breadcrumb trail of transcriptional memory provides a framework for understanding how heterozygous loss-of-function mutations in histone-modifying enzymes may cause severe neurodevelopmental disorders.

## Cotranscriptional Histone Methylation Acts as a Transcriptional Memory

The genome is packaged as chromatin by wrapping DNA around a nucleosome core comprised of histone proteins [1]. Like beads on a string, the spacing of nucleosomes controls access to the genome and is regulated by adding or removing post-translational modifications to histone tails [1]. Some modifications are associated with open chromatin and gene expression, like dimethylation of histone H3 at lysine 4 (H3K4me2), H3K36me3, and H3K79me [2]. Because they are commonly found at sites of active transcription, it is tempting to think that these modifications initiate transcription. However, these particular active histone modifications are only acquired during RNA polymerase (Pol) II elongation. We highlight here some of the evidence supporting H3K4me2 being cotranscriptionally acquired. However, similar evidence suggests that H3K36me3 and H3K79 methylation are also acquired cotranscriptionally [3–8]. In *Saccharomyces cerevisiae* the H3K4 methyltransferase Set1/COMPASS (complex of proteins associated with Set1) associates with RNA Pol II to deposit H3K4 methylation at loci during transcription [9]. Evidence for the **cotranscriptional** (see Glossary) acquisition of H3K4me2 can be also be found in *Drosophila* S2 cells, where the pattern of genomic H3K4me2 is nearly identical to the occupancy of elongating RNA Pol II [10]. Furthermore, the cotranscriptional role of Set1 is conserved in other eukaryotes, where Set1 homologs associate with **COMPASS-like complexes** (called MLL complexes in mammals) [11].

If certain histone modifications are only acquired in the process of RNA Pol II elongation, why then bother adding active modifications at all? In the short term, coupling histone modification to transcription could ease the burden for future transcription. Active modifications help recruit **ATP-dependent chromatin (or nucleosome) remodelers**. In turn, these remodelers increase the spacing of nearby nucleosomes, which can then facilitate further RNA Pol II elongation [12]. This self-reinforcing cycle may help maintain transcription at certain loci over time [13]. However, in the long term, coupling histone modification to transcription may also have an additional epigenetic function: maintaining gene expression patterns through cell division.

## The Breadcrumb Model: Transcriptional Memory Aids in the Coordinate Maintenance of Cell Fate but Poses a Problem for Fate Transitions

**Figure 1.**
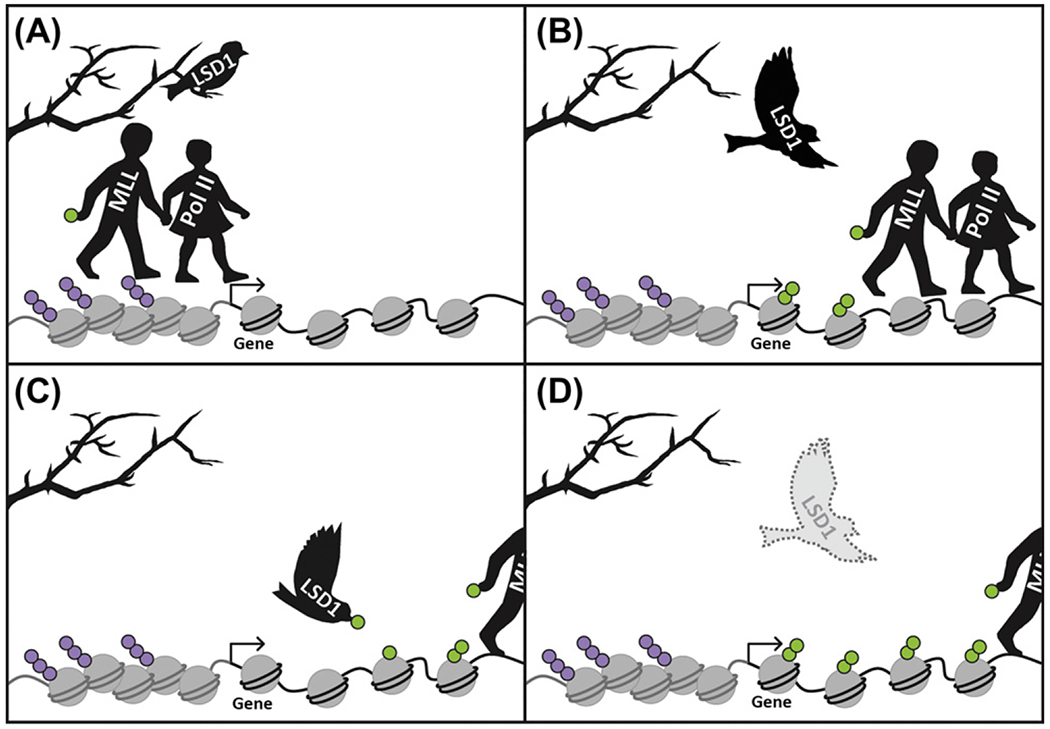
The Breadcrumb Model of Transcriptional Memory (A) During transcription of a gene (black curved line), Pol II and associated histone methyltransferases, like those found in the MLL/COMPASS complex [complex of proteins associated with *S*et1 (called MLL complexes in mammals)], gain access to the transcription start site (designated by arrow). Repressive histone modifications (in purple) keep chromatin in a condensed state and prevent access to DNA (gray curved line). (B) As transcription proceeds, histone methyltransferases add active histone modifications (in green) to nearby nucleosomes. Like a trail of breadcrumbs left by Hansel and Gretel in the fairy tale, these modifications indicate regions of prior transcription. Histone methylation can be inherited through cell divisions, acting as transcriptional memory from mother to daughter cell to facilitate future expression of the locus. (C) When a cell switches transcriptional programs, this memory interferes with proper development. Therefore, during certain important fate transitions, epigenetic reprograming factors like LSD1 erase the breadcrumb trail by removing transcriptional memory. (D) Failures in epigenetic reprogramming (represented by dashed LSD1) result in the inappropriate retention of active modifications. The expression of these loci often has detrimental effects for cell fate.

Here, we describe the breadcrumb model, inspired by the German fairy tale ‘Hansel and Gretel’ (Figure 1, Key Figure) [14]. In the story, a woodcutter’s family is so poor that it can barely feed itself. In desperation, the parents decide to abandon their children, Hansel and Gretel, in the forest to fend for themselves. On the first abandonment, the clever children use white pebbles to leave a trail back to their home; but when they are abandoned for a second time, Hansel and Gretel have only breadcrumbs to use for the trail. The birds of the forest soon peck away the breadcrumbs, leaving the children bereft in the woods. We suggest that active modifications are deposited cotranscriptionally, like a trail of breadcrumbs, to signify which loci have been expressed (Figure 1). To prevent aberrant gene expression, epigenetic reprograming factors may need to remove histone methylation at critical developmental time points in order to erase trails left by prior transcription.

If histone methylation is maintained between cell divisions at a particular locus, it can establish an open chromatin state to facilitate transcription of this locus in each daughter cell. By preserving active chromatin through cell divisions, cells conserve energy by alleviating the need to reactivate genes from scratch. Evidence for this type of transcriptional maintenance can be seen in budding yeast, where patterns of transcriptional activation are maintained through cell division. This maintenance of transcription requires the COMPASS H3K4 methyltransferase complex, and is eliminated by mutating lysine 4 of histone H3 [15]. Furthermore, in both yeast and human cells, the persistence of H3K4me2 enables certain loci that were previously transcribed to be more easily reactivated after multiple cell divisions, through their association with the nuclear pore [16,17]. However, it is important to note that although many histone modifications are associated with gene activation, only a subset function as **transcriptional memory**. For example, H3K4me2 is only acquired in gene bodies during RNA Pol II elongation. This is also likely the case for H3K36me3 and H3K79 methylation. In contrast, H3K4me1 is found at enhancers, and H3K4me3 is largely confined to promoters [8]. At these sites, the acquisition of H3K4me1 and H3K4me3 may be associated with RNA Pol II recruitment but likely does not require RNA Pol II elongation. These distinctions provide an opportunity for the genome to distinguish between loci that are functionally producing mRNAs versus those that are simply poised for future transcription. For example, in certain cases, H3K4me1 and H3K4me3 may function as a transcriptional memory, in a way that is similar to H3K4me2. Examples of H3K4me3 functioning as transcriptional memory include bivalent chromatin during **embryonic stem cell (ESC)** differentiation (see later), or at loci where H3K4me3 spreads over broad domains [18]. In the case of these broad domains, it is possible that H3K4me3 is deposited along with RNA Pol II elongation. However, when H3K4me1 and H3K4me3 are deposited independently of RNA Pol II elongation, they may be associated with the maintenance of transcription over time, or just a consequence of transcription. Thus, the context may determine whether a particular modification functions as transcriptional memory. In support of the latter theory, H3K4me1 has recently been shown not to be required for transcription in ESCs [19]. However, this finding does not preclude a potential role during differentiation, or the possibility that the function of H3K4me1 *in vivo* may differ from its role in cultured ESCs.

The breadcrumbs of transcriptional memory also provide another, perhaps more important, advantage. Cell fates are often initiated by a precise combination of specific transcription factors acting together. Coupling histone methylation to transcription encodes a genome-wide transcriptional maintenance program with just a handful of histone modifications. Therefore, transcriptional memory allows a specific fate to be coordinately maintained in cells descended from the same progenitors, even in the absence of the original initiating transcription factors. Over time, through cell divisions, or even across generations, additional transcription can then further reinforce the pattern of gene regulation. Evidence for the role of transcriptional memory in maintaining cell fate can be found in *Xenopus* cloning experiments performed by Ng and Gurdon [20]. Embryos derived from cloning via somatic cell nuclear transfer (SCNT) often inappropriately express genes from the original tissue that provided the somatic nucleus. This transcriptional memory of cell fate could not be maintained when lysine 4 of histone H3 was mutated, suggesting that the memory may be contained in the methylation of that residue [20]. In addition, they showed that inappropriately retained H3K4 methylation inhibited the efficiency of cloning via SCNT [21].

An elegant example of how the breadcrumb model contributes to cell fate can also be found early in *Drosophila melanogaster* embryonic development. Within a few dozen nuclear division cycles, the identity of future body segments is established through the sequential activation of segmentation genes, which encode different classes of transcription factors. Gap gene proteins are expressed first, which then activate pair-rule genes, which in turn activate segment polarity genes [22]. Segment identity is determined by the combination of specific gap and pair-rule transcription factors present in the segment’s progenitor cells. In *Drosophila*, the COMPASS-like H3K4 methylation complex is known as the **Trithorax group (trxG) complex** [23]. As segmentation genes are being expressed, Trithorax adds active H3K4me to reinforce an open chromatin state. After segmentation, when the original transcription factors are no longer present, an open chromatin state helps to maintain segment identity. Without Trithorax, the absence of H3K4me transcriptional memory causes the loss of segment identity. This loss of cellular fate manifests as a dramatic homeotic transformation of segments later in development [24].

Trithorax’s activity is opposed by the **Polycomb group (PcG)** methyltransferase complex, which adds repressive H3K27me3 at silenced genes [24]. At any one specific locus, H3K4me tends to exclude H3K27me3, and vice versa. However, both modifications do coexist at some enhancers and promoters of important developmental genes. These regions of chromatin, called bivalent loci, help keep genes in stasis: H3K27me3 maintains an inactive state that persists throughout cell divisions, but the presence of H3K4me allows for rapid expression once the H3K27me3 is removed [25,26]. Thus, the presence of repressive H3K27me3 enables the breadcrumbs of transcriptional memory to be maintained without requiring further transcription. This additional layer of regulation is particularly important in stem and progenitor cell populations, which are poised to rapidly choose between renewal versus differentiation into multiple lineages [27].

Although transcriptional memory helps coordinate cell fates during development, it also presents a significant problem during fate transitions (Figure 1). For example, during fertilization, differentiated gametes need to completely reset their genomes to create a totipotent embryo. Stem cells face a similar problem in reverse, since they must swap multipotency for commitment to a specific lineage. Finally, even cells that have already attained terminal differentiation may face the continuous pressure of blocking an inappropriate reacquisition of multipotency. This review focuses on the role of histone methylation during cell fate transitions and how its misregulation in mammals compromises cell fates. We demonstrate how, in each of these three examples, the failure to sweep away the breadcrumbs can leave a false trail of transcription that ultimately contributes to disease.

## Example One: Maternal Factors Reprogram the Epigenome at Fertilization to Ensure Proper Development

Sexual reproduction occurs when two gametes fuse to form a single-celled embryo. But because gametes are the product of a specialized developmental program, their genome bears a distinct transcriptional memory that is incompatible with embryonic development. The embryo is therefore faced with the task of repressing gametic factors while at the same time priming its genome for transcriptional plasticity. To accomplish both goals, the zygotic genome undergoes an extensive reprograming event shortly after fertilization. Reprogramming is accomplished primarily by maternal factors deposited in the oocyte, including histone modifiers. At recently expressed genes, they remove active modifications to prevent the re-expression of gametic genes, or deposit repressive modifications to ensure that these genes stay off (Figure 1C) [28]. For example, in the nematode *Caenorhabditis elegans*, the histone **lysine demethylase** SPR-5 plays an important role in epigenetic reprogramming. SPR-5 is a homolog of mammalian LSD1 (also called KDM1A or AOF2) that removes active H3K4me1/2 from genes previously expressed in the germline, including spermatogenesis genes [29]. When SPR-5 is missing, H3K4me2 is inappropriately retained. After one generation without SPR-5, mutants experience only a slight increase of H3K4me2 in the genome, which has little effect on transcription. However, these higher levels of H3K4me2 are heritable between generations. In the continued absence of SPR-5, more H3K4me2 accumulates with each generation, until eventually enough is amassed to cause misregulation of gametic gene expression. Over a period of twenty generations, this gradual misregulation of gene expression causes *spr-5* mutants to become progressively more sterile [29].

In a similar manner, worms lacking the H3K9 **lysine methyltransferase** MET-2 (a homolog of mammalian SETDB1, which is also called KMT1E or ESET) gradually become sterile over twenty generations [30]. Sterility is caused by the loss of repressive H3K9me2, which indirectly leads to the gradual accumulation of H3K4me2 [31]. In a single generation, neither *spr-5* nor *met-2* single mutants experience significant development defects, which is a testament to the resilience of epigenetic reprogramming. However, animals that lack both SPR-5 and MET-2 experience a substantial postembryonic developmental delay and become sterile within a single generation [31]. These striking phenotypes indicate that, even though reprogramming can handle either the loss of repressive H3K9me2 or the accumulation of active H3K4me2, it cannot tolerate the loss of two complementary factors.

Development in *C. elegans* has the unusual feature of being invariant between individuals, which might allow it to proceed despite reprogramming errors. Conversely, in mammals, the loss of maternal reprogramming factors has much more serious consequences, as summarized in Table 1. Oocytes lacking the SPR-5 homolog LSD1 produce zygotes that fail to activate their zygotic genome and consequently die by the two-cell stage [32,33]. Similarly, oocytes lacking the MET-2 homolog SETDB1 produce zygotes that develop slowly and die by the blastocyst stage [34,35]. Thus, mammalian embryogenesis appears to be more sensitive to the inappropriate retention of transcriptional memory. Intriguingly, partial loss of LSD1 in mouse oocytes gives rise to an occasional surviving pup that, upon adulthood, exhibits obsessive behavioral defects [32]. The presence of adult phenotypes indicates that the failure to reprogram a single-cell embryo has consequences that can persist throughout many cell divisions and fate changes.

The consequences of failing to reprogram transcriptional memory are severe enough that one wonders why embryos don’t simply erase all prior histone modifications. However, several studies have highlighted the importance of balancing the erasure of transcriptional memory with its retention. In mice, maternal mutations in either the H3K4 methyltransferase KMT2B or the H3K36 methyltransferase SETD2 cause a two-cell arrest that resembles the loss of LSD1 or SETDB1 [36,37]. This similarity suggests that some epigenetic transcriptional memory needs to be maintained to allow for normal embryogenesis to proceed. Another example of this requirement can be found in worms. During gametogenesis, germline-specific genes are expressed and acquire active H3K36me3 through the activity of the transcription-coupled H3K36 methyltransferase MET-1 [30,38]. However, little transcription occurs during early embryogenesis, particularly in germline precursor cells, where the maternal factor PIE-1 blocks Pol II elongation [39]. The transcriptional memory of H3K36me3 is instead maintained by the activity of a transcription-independent H3K36 methyltransferase, MES-4. Because MES-4’s activity does not rely on transcription, it can help preserve transcriptional memory during the crucial developmental window of early embryogenesis, when no transcription occurs [38,40]. Worms that lack MES-4 are maternal-effect sterile because they lose H3K36me3 transcriptional memory at germline-specific genes and fail to reactivate the germline [38].

## Example Two: Epigenetic Reprogramming Factors Poise ESCs for Successful Differentiation

A stem cell has two opposing goals, each with its own transcriptional program: it must proliferate to renew the pool of stem cells, but it must also be ready to differentiate into a specific lineage [41]. Transcriptional memory driven by **pluripotency factors** helps stem cells to maintain a state of self-renewal, while epigenetic reprogramming removes this memory to allow differentiation to proceed unimpeded, as summarized in Table 2 [27]. One example of this fate switching can be found in mammalian ESCs, which are derived from the inner cell mass of blastocyst-stage embryos. ESCs are pluripotent, meaning that they are poised to differentiate into any of the three possible germ layers. Like bivalent loci in *Drosophila* embryos, genes that are required for lineage commitment are maintained in a poised state by the presence of both active H3K4me3 and repressive H3K27me3 at *cis*-regulatory regions [26]. Upon differentiation, H3K27me3 is removed at loci involved in lineage commitment [25]. This removal of H3K27me3 resolves **bivalent domains** into activating domains, enabling the remaining H3K4me3 to promote transcription.

Similar to what occurs during fertilization, the breadcrumbs of transcriptional memory must also be removed during ESC differentiation. During this process, the resolution of bivalent domains is accompanied by silencing the pluripotency transcriptional program. ESC pluripotency is maintained by a transcriptional network, driven by key factors like OCT4, SOX2, KLF4, and NANOG [42]. These transcription factors are strongly expressed and acquire high levels of H3K4me transcriptional memory at their promoters and enhancers. In addition, each of the pluripotency transcription factors induces its own expression as well as the expression of the other pluripotency factors. This feedback creates a self-reinforcing loop that tilts the balance in ESCs towards maintenance of a pluripotent state [42]. After differentiation is induced, LSD1 decommissions pluripotency factors by removing H3K4me transcriptional memory at their enhancers and promoters. Without LSD1 to decommission the pluripotency network, transcription of the critical pluripotency factors is inappropriately maintained alongside the differentiation program. This dual identity causes cell death soon after differentiation is induced [43]. A similar process is required during the differentiation of hematopoietic stem cells, and likely many other types of stem cells [44]. Consistent with these defects, mice lacking LSD1 die before specification of germ layers occurs at embryonic day eight [45,46].

Epigenetic reprogramming during stem cell differentiation (Table 2) resembles epigenetic reprogramming at fertilization (Table 1) in multiple ways. For example, at fertilization the removal of active H3K4me is accompanied by the deposition of repressive H3K9me. This also occurs in ESCs, though in ESCs the addition of H3K9me may occur primarily through the methyltransferase G9a (also called Kmt1c or Ehmt2), rather than SETDB1 [47–49]. Similar to fertilization, ESCs may also need to balance reprograming with the retention of some transcriptional memory. For example, ESCs that lack the transcription-coupled methyltransferases KMT2D (which adds H3K4me) or SETD2 (which adds H3K36me) also have severe differentiation defects [50,51].

## Example Three: Transcriptional Memory Must Be Continuously Suppressed, Even in Differentiated Cells

Spurious transcription occurs throughout the genome and is associated with the deposition of active histone modifications. This deposition of active histone modifications has the potential to open chromatin and make it accessible to transcriptional machinery. This is particularly true in self-reinforcing networks, such as the pluripotency network, where initial transcription can lead to further transcription and the increasing accumulation of active transcriptional memory. Thus, the coupling of active histone modifications to transcription imposes a perpetual risk of reactivating prior developmental programs. As with reprogramming after fertilization, the failure to contend with this transcriptional memory may contribute to specific diseases. For example, although LSD1 has predominantly been shown to function during key developmental transitions, in adult mice it is still expressed broadly across many tissues [52]. What might be the role of a memory eraser in cells that do not expect any future fate transitions?

One indication comes from examining the conditional loss of LSD1 in adult mice. Within days of losing LSD1 protein, adult mice experience major neuronal cell death and soon resemble late-stage Alzheimer’s disease models. This neurodegeneration is associated with significant inappropriate gene activation, including the reactivation of the pluripotency factors KLF4 and OCT4. This suggests that neurons have a continuous requirement for LSD1 to suppress transcriptional memory and prevent neurodegeneration [52]. Thus, in some cases transcriptional memory may require constant reprogramming. Remarkably, in patients that suffer from Alzheimer’s disease, LSD1 inappropriately associates with tau protein aggregates in the cytoplasm [52]. This finding raises the intriguing possibility that pathological protein aggregates may interfere with the suppression of transcriptional memory and contribute to Alzheimer’s disease. Although LSD1 is currently the only known example of how a histone-modifying enzyme is continually employed to suppress transcriptional memory, the regulation of transcriptional memory in terminally differentiated cells is not unlike the regulation of transcriptional memory at fertilization and in stem cells (Tables 1 and 2). As a result, it is likely that other histone modifiers (like those that add repressive H3K9me or active H3K36me) will also be found to be required in differentiated cells.

## Defects in Epigenetic Reprogramming Contribute to Additional Diseases

As highlighted by these three examples, it is clear that histone-modifying enzymes are important for key developmental transitions. Thus, it is not surprising that mutations in several of the histone-modifying enzymes that have been discussed here are associated with neurodevelopmental disorders that are characterized by intellectual disability and developmental delay. For example, mutations in the H3K27 demethylase UTX or the H3K4 methyltransferase KMT2B cause **Kabuki syndrome** [53], and mutations in the H3K36 methyltransferase NSD1 cause **Sotos syndrome** [54]. Additionally, three patients have been discovered with mutations in LSD1, and their symptoms strongly resemble Kabuki syndrome [55,56]. Interestingly, the alleles that these patients carry are dominant, so these patients still carry a normal copy of the affected enzyme. How could the loss of only one copy of a histone-modifying enzyme give rise to a devastating neurodevelopmental disorder? Having only a single wild-type copy could cause a null phenotype, a hypomorphic phenotype, or create a sensitized background for other effects. The first two models are scenarios where the histone-modifying enzyme is haploinsufficient. In the first possibility, losing a single copy results in a complete loss of function for the enzyme. In support of this possibility, many histone-modifying enzymes have been proposed to be part of coexpressed regulatory networks. The complexity of this type of coregulatory network may magnify the effects of losing only one copy of a histone-modifying enzyme [57]. However, this explanation is difficult to reconcile with the phenotypes observed in mouse models of these neurodevelopmental disorders. The maternal loss of KMT2D, NSD1, LSD1, or UTX, each causes a very early embryonic arrest (Table 1). In addition, complete zygotic loss of KMT2D, NSD1, or LSD1 is not viable, and zygotic loss of UTX is only partially viable [45,58–60]. Furthermore, loss of KMT2D, LSD1, or UTX in ESCs results in severe proliferation and/or differentiation defects (Table 2). Therefore, if a heterozygous loss-of-function mutation in a human patient resulted in a complete loss of function, it seems unlikely that the embryo would survive. It is possible that the function of these histone-modifying enzymes in humans is inherently different, but it is more plausible that the mutant allele in these neurodevelopmental disorders is haploinsufficient without being a complete null. In support of this second possibility, heterozygous *KMT2D* mutant mice are viable but display some defects that are reminiscent of Kabuki syndrome patients [59]. In this case, cells may have enough enzymatic activity to fulfill the requirements for activity (like for the maternal requirement, or in ESCs), but the remaining activity is insufficient in cell lineages that give rise to symptoms associated with the disorder. However, it is difficult to reconcile how the loss of one allele could achieve precisely this level of disruption in each of these neurodevelopmental disorders.

A third possibility is that the loss of one allele is not severe enough to cause a neurodevelopmental disorder on its own. Consistent with this possibility, researchers have discovered two pedigrees with hypomorphic mutations in LSD1. These families suffer from increased susceptibility to leukemia, due to somatic loss of the normal copy of LSD1, but these patients were not reported to have neurodevelopmental disorders [61]. It is possible that neurodevelopmental symptoms were simply left unreported in these patients. However, if these leukemia patients do not have neurodevelopmental symptoms, their cases would suggest that a partial loss of LSD1 function is not sufficient to cause a Kabuki-like syndrome. Results from UTX mice also suggest that heterozygous loss-of-function mutations are not sufficient to generate phenotypes. Mice with neural crest mutations in UTX only have craniofacial defects when both copies of UTX are lost, rather than just one [58]. This result suggests that in mice, UTX function must be fully compromised to generate phenotypes that resemble those seen in Kabuki syndrome patients.

Based on these considerations, we propose an alternative two-hit hypothesis of epigenetic reprogramming to explain how heterozygous loss-of-function mutations can cause neurodevelopmental defects. Similar to the two-hit hypothesis of cancer causation, it is possible that defects in patients with heterozygous loss-of-function mutations in histone-modifying enzymes may arise from a population that is additionally destabilized for reprogramming [62]. At key developmental transitions, like fertilization or stem cell differentiation, the loss-of-function mutation may combine with other defects to perturb reprogramming past the point of recovery. These other defects that make patients susceptible may arise from stochastic differences, genetic background, effects of maternal age, or other environmental factors. For example, certain oocytes may have slightly less SETDB1, perhaps due to advanced maternal age. Normally, this reduction would have no effect, but if an oocyte also has a genetic background that reduces the expression of LSD1, or receives an environmental insult that impinges upon LSD1, the two defects could act synergistically during reprogramming after fertilization to drastically impact developmental success. This could give rise to defects that are similar to what is observed when LSD1 is hypomorphic maternally in mice [32].

Regardless of whether loss-of-function mutations in histone-modifying enzymes are haploinsufficient, or whether they require an additional defect to cause disease, it seems likely that their consequences are propagated by the breadcrumbs of transcriptional memory. In neurodevelopmental syndromes like Kabuki or Sotos syndrome, there are certain cell lineages that appear to be consistently affected. For example, it is likely that defects in neural crest give rise to the craniofacial defects in these patients. There are two possibilities that could explain these lineage defects. The first possibility is that every cell within the affected lineages is independently affected by the loss-of-function mutation. However, in ESCs, mutations that cause Kabuki or Sotos syndrome have very little effect until the ESCs begin to differentiate (Table 2). This finding suggests that these enzymes are not required unless cells undergo a transition in cell fate. The second possibility is that mutations in histone-modifying enzymes only have an effect at key developmental transitions, when cells choose between distinct cell fates; subsequently, the inappropriate retention of histone methylation functions as a false breadcrumb trail in the descendent of these cells. For example, Kabuki syndrome can be caused by mutations in either the H3K4 methyltransferase or the H3K27 demethylase, which are required to resolve poised bivalent chromatin domains to active domains. It could be that Kabuki syndrome is caused by a failure to resolve bivalent chromatin during critical reprogramming windows, such as maternal reprogramming, blastocyst differentiation, or neural crest differentiation. The inappropriately programmed chromatin would then be propagated in the resulting tissue as breadcrumbs of transcriptional memory.

## Concluding Remarks and Future Perspectives

In the past decade, it has become clear that histone methylation can persist through cell divisions. We propose that these breadcrumbs of transcriptional memory provide both a clear benefit and a subsequent burden. Transcriptional memory allows cells to reactivate transcription easily and coordinate developmental programs during differentiation. But after cells transition to a new fate, this transcriptional memory must be actively removed to prevent inappropriate gene expression. As illustrated in this review, the failure to reprogram the genome at key points in development can lead to disease. As a result, multiple chromatin modifiers must coordinate their activity to regulate the breadcrumbs of transcriptional memory and prevent disease.

The resilience of epigenetic reprogramming at critical developmental transitions may obscure the full contributions of individual chromatin modifiers (see Outstanding Questions). In particular, it is difficult to reconcile how mutations in a single allele of a histone-modifying enzyme can cause devastating neurodevelopmental disorders. We propose a way to think about this problem inspired by the two-hit hypothesis of carcinogenesis. During reprogramming, loss of one copy of a histone-modifying enzyme combines with a second defect in either: (i) another histone-modifying enzyme, (ii) the genetic background, or (iii) some type of environmental exposure. If reprogramming diseases do arise from such a multifaceted etiology, it will be difficult to determine unique contributions of the factors involved. Thus, it may be fruitful to use sensitized genetic backgrounds to investigate the effects of null and hypomorphic alleles. Additionally, because defects may have to occur in two, or even three modifiers before phenotypes appear, it would likely be helpful to screen for phenotypes in genetically tractable models like *C. elegans* or *Drosophila*. Regardless, the two-hit hypothesis of epigenetic reprogramming predicts that loss of one allele of a histone-modifying enzyme should not be sufficient to induce the corresponding human diseases. This prediction can be directly tested. As researchers sequence more human genomes, there should be nonsyndromic patients that have alleles that resemble those found in syndromic patients. The existence of these otherwise hidden alleles would strongly support a model where loss of one allele combines with other defects to synergistically perturb reprogramming.

## Figures and Tables

**Table 1.** The Maternal Effect of Chromatin Modifier Mutations in Mice

Name (complex)	Alternative names	Activity	M−Z+ molecular effect	M−Z+ progeny effect	Refs
*Setd1b*		HMT H3K4me2/3	No change in H3K4me; transcriptional upregulation caused by aberrant repression of transcription factors	Polyspermy; arrest at one to two-cell stage	Brici *et al.* (2017) [63]
*Kmt2d*	*Mll2, Mll4*	HMT H3K4me2/3	Reduced H3K4me2/3; transcriptional misregulation	Arrest at one to two-cell stage	Andreu-Vieyra *et al.* (2010) [37]
*Setd2*		HMT H3K36me3	Decreased H3K36me2; spread of H3K4me3 and H3K27me3; aberrant H3K4me3 at imprinting control regions; loss of DNA methylation	Arrest at one-cell stage	Xu *et al.* (2019) [36]
*G9a*	*Ehmt2, Kmt1c*	HMT H3K9me1/2	Loss of H3K9me2; transcriptional upregulation at four-cell stage	Developmental delay; some arrest of preimplantation embryos	Zylicz *et al.* (2018) [64] and Au Yeung *et al.* (2019) [65]
*Setdb1*	*Eset, Kmt1e*	HMT H3K9me	Reduced H3K9me2/3; repetitive element upregulation; aberrant meiosis; aberrant oocyte transcription	Developmental delay; arrest by blastocyst stage	Kim *et al.* (2016) [34] and Eymery *et al.* (2016) [35]
*Ezh2* (PRC2)		HMT H3K27me2/3	Reduced H3K27me and H3K9me	Reduced body weight at birth	Erhardt *et al.* (2003) [66]
*Eed* (PRC2)		HMT H3K27me2/3	Reduced H3K27me3 until eight-cell stage; biallelic expression of imprinted genes	Developmental delay, male-biased lethality	Inoue *et al.* (2018) [67] and Prokopuk *et al.* (2018) [68]
*Ring1* (PRC1)	*Ring1a*	HMT H3K27me3	*Ring1; Rnf2* double mutants have transcriptional misregulation	*Ring1; Rnf2* double mutants arrest at two-cell stage	Posfai *et al.* (2012) [69]
*Rnf2* (PRC1)	*Ring1b*	HMT H3K27me3	Single mutants had no effect; *Ring1; Rnf2* double mutants have transcriptional misregulation	*Ring1; Rnf2* double mutants arrest at two-cell stage	Posfai *et al.* (2012) [69] and Terranova *et al.* (2008) [70]
*Kdm1a*	*Lsd1, Aof2*	KDM H3K4me1/2	Accumulation of H3K4me and H3K9me; loss of DNA methylation at **CpG islands**; failed zygotic genome activation	Arrest at one to two-cell stage; rare survivors have behavioral abnormalities	Wasson *et al.* (2016) [32], Ancelin *et al.* (2016) [33], and Stewart *et al.* (2015) [71]
*Kdm1b*	*Lsd2, Aof1*	KDM H3K4me1/2	Increased H3K4me; loss of DNA methylation at CpG islands; loss of imprinting	Lethality by mid-gestation	Stewart *et al.* (2015) [71] and Ciccone *et al.* (2009) [72]
*Kdm6a*	*Utx*	KDM H3K27me3	siRNA knockdown of both *Kdm6a* and *Kdm6b* caused increased H3K27me3	siRNA knockdown of both *Kdm6a* and *Kdm6b* impaired preblastocyst development	Yang *et al.* (2016) [73]
*Kdm6b*	*Jmjd3*	KDM H3K27me3	In cows, oocyte siRNA caused increased H3K27me3 and transcriptional misregulation	Bovine oocyte siRNA impaired preblastocyst development	Chung *et al.* (2017) [74]
*Brg1* (SWI/SNF)	*Smarca4*	Chromatin remodeler	Reduced H3K4me2; transcriptional downregulation; failed zygotic genome activation	Arrest by two to four-cell stage	Bultman *et al.* (2006) [75]
*Trim28*	*Kap1, Tif1β*	Recruits CHD3 and SETDB1	Aberrant imprinting	Postimplantation lethality	Messerschmidt *et al.* (2012) [76]

**Table 2.** The Effect of Mutations or Knockdown of Chromatin Modifier in Mammalian ESCs

Name (complex)	Alternative names	Activity	ESC origin	Molecular effect	ESC effect	Refs
*Wdr5* (MLL core)	*Swd3, Cfap89*		Mouse	Reduced H3K4me3; reduced pluripotency gene expression; upregulation of differentiation genes	Reduced self-renewal; enhanced differentiation	Ang *et al.* (2011) [77]
*Dpy30* (MLL core)			Mouse	Reduced H3K4me3 at developmental genes; impaired expression of developmental genes	No effect on self-renewal; impaired differentiation (neural)	Jiang *et al.* (2011) [78]
*Rbbp5* (MLL core)	*Swd1*		Mouse	Reduced H3K4me3 at developmental genes; impaired expression of developmental genes	No effect on self-renewal; impaired differentiation (neural)	Jiang *et al.* (2011) [78]
*Mll1* (MLL)	*Kmt2a*	KMT H3K4me	Mouse	No effect on H3K4me3; little effect on overall transcription		Denissov *et al.* (2014) [79], Zhang *et al.* (2016) [80]
*Mll2* (MLL)	*Kmt2b, Wbp7*	KMT H3K4me2/3	Mouse	Reduced H3K4me3 (especially bivalent gene promoters); increased occupancy of PRC2; increased H3K27me3; aberrant transcription during differentiation	No effect on self-renewal; impaired differentiation (all germ layers)	Denissov *et al.* (2014) [79], Lubitz *et al.* (2007) [81], Glaser *et al.* (2009) [82], Hu *et al.* (2013) [83], Mas *et al.* (2018) [84]
*Mll4* (MLL)	*Kmt2d, Alr*	KMT H3K4me1/2	Mouse	Reduced H3K4me1 at enhancers; *Mll3; Mll4* double mutants had reduced H3K4me1/2 at target enhancers, reduced H3K27ac, gene misregulation during differentiation	No effect on self-renewal; impaired differentiation; *Mll3; Mll4* double mutants had impaired differentiation	Dorighi *et al.* (2017) [19], Wang *et al.* 2016 [51], Rickels *et al.* (2017) [85], Cao *et al.* (2018) [86]
*Mll3* (MLL)	*Kmt2c*	KMT H3K4me1/2	Mouse	*Mll3; Mll4* double mutants had reduced H3K4me1/2 at target enhancers, reduced H3K27ac, gene misregulation during differentiation	No effect on self-renewal; *Mll3; Mll4* double mutants had impaired differentiation	Dorighi *et al.* (2017) [19], Wang *et al.* (2016) [51], Rickels *et al.* (2017) [85]
*Nsd1*		KMT H3K36me2	Mouse	Reduced H3K36me2; increased H3K27me3 mediated by PRC2		Streubel *et al.* (2018) [87]
*Setd2*	*Kmt3a*	KMT H3K36me3	Mouse	Reduced H3K36me3	No effect on self-renewal; impaired differentiation (endoderm)	Zhang *et al.* (2014) [50]
*Setd1A* (Set1A)	*Kmt2f, Set1a*	KMT H3K4me	Mouse	Reduced H3K4me (independent of SET domain); downregulation of pluripotency genes; upregulation of differentiation genes	Impaired self-renewal; impaired proliferation; impaired differentiation	Bledau *et al.* (2014) [88], Fang *et al.* (2016) [89], Sze *et al.* (2017) [90]
*Setd1B* (Set1B)		KMT H3K4me	Mouse	No effect on H3K4me	No effect	Bledau *et al.* 2014 [88]
*Cxxc1* (Set1A & SET1B)	*Cfp1, Cgbp*	Binds unmethylated CpG	Mouse	Reduced H3K4me3 at highly-expressed gene promoters; no effect on transcription; ectopic H3K4me3 and increased expression of nearby genes; reduced CpG methylation	No effect on self-renewal; impaired differentiation	Carlone *et al.* (2005) [91], Butler *et al.* (2008) [92], (Butler) *et al.* (2009) [93], Clouaire *et al.* (2012) [94]
*Setd7*	*Kmt7, Set7, Set9*	KMT H3K4me1	Mouse	Reduced H3K4me1, downregulation of endoderm genes		Tuano *et al.* (2016) [95]
			Human	Reduced H1 incorporation and reduced silencing of pluripotency genes	Delayed differentiation	Castano *et al.* (2016) [96]
*G9a* (with *Glp*)	*Kmt1c, Ehmt2*	KMT H3K9me1/2	Mouse	Reduced H3K9me2/3; reduced CpG methylation at target gene promoters, but no derepression	Impaired differentiation	Tachibana *et al.* (2008) [48], Feldman *et al.* (2006) [49], Dong *et al.* (2008) [97]
			Human	Reduced H3K9me3	No effect on self-renewal; impaired differentiation	Tachibana *et al.* (2002) [98]
*Glp* (with G9a)	*Kmt1d, Ehmt1*	KMT H3K9me3	Mouse	Reduced H3K9me1/2; reduced CpG methylation at promoters of target genes, but no derepression; derepression of pluripotency genes	Impaired differentiation	Tachibana *et al.* (2005) [47], Tachibana *et al.* (2008) [48], Liu *et al.* (2015) [99]
*Setdb1*	*Kmt1e, Eset*	KMT H3K9me2/3	Mouse	Reduced H3K9me3; downregulation of pluripotency genes; derepression of bivalent genes; upregulation of trophoblast genes and repetitive elements	Impaired self-renewal; aberrant differentiation to trophectoderm	Bilodeau *et al.* (2009) [100], Yeap *et al.* (2009) [101], Yuan *et al.* (2009) [102], Matsui *et al.* (2010) [103], Lohmann *et al.* 2010 [104]
*Suv39h1*	*Kmt1a*	KMT H3K9me3	Mouse	*Suv39h1; Suv39h2* double mutants had reduced H3K9me3 at repetitive elements		Bulut-Karslioglu *et al.* (2014) [105]
*Suv39h2*	*Kmt1b*	KMT H3K9me3	Mouse	*Suv39h1; Suv39h2* double mutants had reduced H3K9me3 at repetitive elements		Bulut-Karslioglu *et al.* (2014) [105]
*Ezh1*		KMT H3K27me	Mouse	No effect on H3K27me; *Ezh1; Ezh2* double mutants phenocopy Eed mutants		Shen *et al.* (2008) [106]
*Ezh2* (PRC2)		KMT H3K27me2/3	Mouse	Reduced H3K27me3 (but not at PcG target gene promoters)	No effect on self-renewal; slightly impaired differentiation	Shen *et al.* (2008) [106]
			Human	Reduced H3K27me; loss of H3K27me3 at promoters and derepression of PcG targets	Impaired self-renewal, proliferation, and differentiation	Collinson *et al.* (2016) [107]
*Eed* (PRC2)		KMT H3K27me2/3	Mouse	Reduced H3K27me; loss of H3K27me3 at PcG target gene promoters; derepression of bivalent or PcG target genes (especially neural)	No effect on self-renewal or proliferation; impaired differentiation	Shen *et al.* (2008) [106], Montgomery *et al.* (2005) , Azuara *et al.* (2006) , Schoeftner *et al.* (2006) , Boyer *et al.* (2006) [111]
*Suz12* (PRC2)		KMT H3K27me2/3	Mouse	Reduced H3K27me2/3; upregulation of differentiation genes	No effect on proliferation; impaired differentiation	Pasini *et al.* (2007) [112]
			Human	Reduced H3K27me3 and H3K9me3		de la Cruz *et al.* (2007) [113]
*Jarid2* (PRC2)		Noncatalytic	Mouse	No effect on H3K27me3; upregulation of PcG target genes	No effect on proliferation	Peng *et al.* (2009) [114]
*Ring1B* (PRC1)		KMT H3K27me3	Mouse	No effect^a^; reduced H2Aub1; upregulation of differentiation genes; *Ring1A; Ring1B* double mutants had loss of H2Aub1, impaired proliferation upregulation of differentiation genes	No effect on self-renewal; impaired differentiation; *Ring1A; Ring1B* double mutants had precocious differentiation	de Napoles *et al.* (2004) [115], Leeb&Wutz (2007) [116], Stock *et al.* (2007) [117], Endoh *et al.* (2008) [118]
*Ring1A* (PRC1)		KMT H3K27me3	Mouse	*Ring1A; Ring1B* double mutants had loss of H2Aub1, impaired proliferation upregulation of differentiation genes	*Ring1A; Ring1B* double mutants had precocious differentiation	Stock *et al.* (2007) [117], Endoh *et al.* (2008) [118]
*Dot1L*	*Kmt4*	KMT H3K79me	Mouse	Reduced H3K79me; reduced H3K9me2 and H4K20me3 at centromeres and telomeres	Impaired proliferation; no effect on proliferation	Jones *et al.* (2008) [119], Barry *et al.* (2009) [120]
*Smyd5*		KMT H4K20me3	Human	Decreased H4K20me3 and H3K9me3; upregulation of differentiation genes and repetitive elements	Impaired self-renewal; impaired differentiation	Kidder *et al.* (2017) [121]
*Lsd1*	*Kdm1a, Aof2*	KDM H3K4me1/2	Mouse	Little effect on global H3K4me; increased H3K4me2/3 at promoters and enhancers of target genes; decreased H3K9me2 at promoters of target genes; derepression of pluripotency genes during differentiation	No effect on self-renewal; impaired proliferation; no effect on proliferation; impaired differentiation into extraembryonic tissues; death upon differentiation	Whyte *et al.* (2012) [43], Wang *et al.* (2009) [46], Foster *et al.* (2010) [122], Macfarlan *et al.* (2011) [123]
			Human	Increased H3K4me2/3 at bivalent genes; upregulation of differentiation genes (mesoderm and endoderm)	Precocious differentiation	Adamo *et al.* (2011) [124]
*Jarid1b*	*Kdm5b, Rbp2, Plu1*	KDM H3K4me2/3	Mouse	Increased H3K4me3; increased cryptic transcription; no cryptic transcription; downregulation of pluripotency genes; derepression of pluripotency genes during differentiation	No effect on self-renewal or proliferation; impaired self-renewal and proliferation; precocious and impaired differentiation (especially ectoderm)	Xie *et al.* (2011) [125], Schmitz *et al.* (2011) [126], Kidder *et al.* (2013) [127]
*Jarid1c*	*Kdm5c, Smcx*	KDM H3K4me3	Mouse	Increased H3K4me3, reduced H3K4me1, and misregulation of target genes		Outchkourov *et al.* (2013) [128]
*Jarid1a*	*Kdm5ba, Rbp2*	KDM H3K4me2/3	Mouse	Increased H3K4me3 at PcG target genes; downregulation of pluripotency genes; upregulation of PcG target genes		Pasini *et al.* (2008) [129], Lin *et al.* (2011) [130]
*Jhdm1b*	*Kdm2b, Fbxl10*	KDM H3K36me2, E3 ligase	Mouse	Decreased H2AK119ub1; reduced PRC1 binding at target genes; upregulation of differentiation genes	No effect on self-renewal or proliferation; impaired differentiation (does not require KDM activity)	He *et al.* (2013) [131], Wu *et al.* (2013) [132]
*Jmjd2a*	*Kdm4a*	KDM H3K9me2/3, H3K36me2/3	Mouse	Increased H3K9me at target genes; downregulation of target genes; *Jmjd2a; Jmjd2c* double mutants had increased H3K9me3 and H3K36me3, gene misregulation	No effect on self-renewal; impaired differentiation; *Jmjd2a; Jmjd2c* double mutants had impaired proliferation and precocious differentiation	Wu *et al.* (2015) [133], Pedersen *et al.* (2016) [134]
*Jmjd2b*	*Kdm4b*	KDM H3K9me2/3, H3K36me2/3	Mouse	Downregulation of pluripotency genes; upregulation of differentiation genes; *Jmjd2b; Jmjd2c* double mutants have increased H3K9me3	Impaired self-renewal; no effect on self-renewal; impaired differentiation	Pedersen *et al.* (2016) [134], Das *et al.* (2014) [135]
*Jmjd2c*	*Kdm4c, Gasc1*	KDM H3K9me3, H3K36me3	Mouse	Increased H3K9me2/3, especially at target genes; little effect on H3K9me3 or H3K36me3; downregulation of pluripotency genes; upregulation of differentiation genes; *Jmjd2a; Jmjd2c* double mutants had increased H3K9me3 and H3K36me3, gene misregulation	Impaired self-renewal; no effect on self-renewal; precocious differentiation; impaired differentiation; *Jmjd2a; Jmjd2c* double mutants had impaired proliferation, and precocious differentiation	Wu *et al.* (2015) [133], Pedersen *et al.* (2016) [134], Das *et al.(*2014) [135], Loh *et al.* (2007) [136], Pedersen *et al.* (2014) [137],Tomaz *et al.* (2017) [138]
*Jmjd1a*	*Kdm3a, Jhdm2a*	KDM H3K9me1/2	Mouse	Increased H3K9me2/3; downregulation of pluripotency genes; upregulation of differentiation genes; *Jmjd1a; Jmjd1b* double mutants have more increased H3K9me2/3 and gene misregulation than either single mutant	Impaired self-renewal and proliferation; precocious differentiation	Loh *et al.* (2007) [136], Kuroki *et al.* (2018) [139]
*Jmjd1b*	*Kdm3b, Jhdm2b*	KDM H3K9me1/2	Mouse	Increased H3K9me2/3; gene misregulation; *Jmjd1a; Jmjd1b* double mutants have more increased H3K9me2/3 and gene misregulation than either single mutant	Impaired self-renewal and proliferation	Kuroki *et al.* (2018) [139]
*Kdm7b*	*Phf8*	KDM H3K9me2	Mouse	Increased H3K9me2	No effect on self-renewal or proliferation; precocious differentiation (mesoderm)	Tang *et al.* (2016) [140]
*Utx*	*Kdm6a*	KDM H3K27me3	Mouse	No effect on global H3K27me3; increased H3K27me3 and decreased H3K4me at target genes and enhancers	No effect on self-renewal or proliferation; impaired differentiation (especially mesoderm, independent of KDM activity)	Wang *et al.* (2012) [141], Lee *et al.* (2012) [142], Welstead *et al.* (2012) [143], Mansour *et al.* (2012) [144], Morales Torres *et al.* (2013) [145], Wang *et al.* (2017) [146]

a Conflicting observations are included with the corresponding reference cited [118].

## Highlights

The breadcrumb model: like a trail of breadcrumbs, histone methylation is added cotranscriptionally to retain a memory of prior gene expression.

The inappropriate retention of histone methylation has developmental consequences during certain cell fate transitions, like immediately after fertilization or during stem cell differentiation.

Transcriptional memory may need to be continuously suppressed, even in terminally differentiated cells.

The two-hit hypothesis of epigenetic reprogramming: weak heterozygous mutations in histone-modifying enzymes combine with a second environmental or genetic defect during vulnerable developmental time points to compromise epigenetic reprogramming.

## Outstanding Questions

What other differentiated cells require continuous reprogramming in mammals? Are the other enzymes involved in reprogramming at fertilization and in ESCs also required for continuous reprogramming in terminally differentiated cells?

How can we identify human patients with maternal contributions to reprogramming diseases (whether genetic or otherwise)? Is there a way to distinguish maternal contributions from zygotic contributions in human diseases?

What other conditions may interact with heterozygous loss-of-function mutations in chromatin-modifying enzymes to cause neurodevelopmental disorders?

How do we identify the synergistic interactions between histone-modifying enzyme networks, between mutations and genetic background, or between mutations and the environment?

## Glossary

ATP-dependent chromatin (or nucleosome) remodelers: ATP-dependent protein complexes that rearrange or eject nucleosomes to control chromatin compaction.
Bivalent domain: a chromatin domain containing both activating (H3K4) and repressing (H3K27) histone methylation simultaneously to poise associated loci for activation or repression during differentiation.
COMPASS-like complex: complex of proteins associated with Set1 (called MLL complexes in mammals): the cotranscriptional H3K4 methyltransferase complex.
Cotranscriptional chromatin modification: histone or DNA modifications that are coupled to the elongation of RNA Pol II.
CpG islands: region of DNA with enriched, unmethylated CpG dinucleotides compared to the rest of the genome; many promoters in the human genome contain CpG islands.
Embryonic stem cells (ESCs): pluripotent stem cells derived from the inner cell mass of blastocyst-stage embryos that can give rise to all three germ layers.
Kabuki syndrome: neurodevelopmental disorder associated with dominant loss of function mutations in histone-modifying enzymes.
Lysine demethylases (KDMs): catalyze the post-translational removal of methyl groups from lysines on histones.
Lysine methyltransferases (KMTs): catalyze the post-translational addition of methyl groups from lysines on histones.
M−Z+: genotype in which the mother or oocyte has a mutation or is depleted for a factor, but the zygote has at least one wild-type allele. This condition is used to determine the maternal effect of a factor.
Pluripotency factors: a set of transcription factors that maintain a state of self-renewal in stem cells.
Polycomb group (PcG): a family of protein complexes that modify chromatin structure to repress gene expression.
Sotos syndrome: neurodevelopmental disorder associated with dominant loss of function mutations in histone-modifying enzymes.
Transcriptional memory: an epigenetic phenomenon whereby transcription of a locus affects its local chromatin environment; in some cases, it may be maintained through cell divisions.
Trithorax group (trxG) complexes: a family of protein complexes that modify chromatin structure to promote gene expression.
